# Probiotic *Lactobacillus sakei* proBio-65 Extract Ameliorates the Severity of Imiquimod Induced Psoriasis-Like Skin Inflammation in a Mouse Model

**DOI:** 10.3389/fmicb.2018.01021

**Published:** 2018-05-17

**Authors:** Irfan A. Rather, Vivek K. Bajpai, Yun Suk Huh, Young-Kyu Han, Eijaz A. Bhat, Jeongheui Lim, Woon K. Paek, Yong-Ha Park

**Affiliations:** ^1^Department of Applied Microbiology and Biotechnology, School of Biotechnology, Yeungnam University, Gyeongsan, South Korea; ^2^Department of Energy and Materials Engineering, Dongguk University, Seoul, South Korea; ^3^Department of Biological Engineering, Inha University, Incheon, South Korea; ^4^Department of Biochemistry, Yeungnam University, Gyeongsan, South Korea; ^5^National Science Museum, Ministry of Science, ICT and Future Planning, Daejeon, South Korea

**Keywords:** psoriasis, skin inflammation, *Lactobacillus sakei*, lactic acid bacteria, imiquimod

## Abstract

This study was designed to evaluate the protective effect of ethanol extract (SEL001) isolated from a potent probiotic strain *Lactobacillus sakei* proBio-65 on imiquimod (IMQ)-induced psoriasis-like skin inflammation in a mouse model. Histopathological and histomorphometrical changes in the ear and dorsal skin tissues were observed under hematoxylin and eosin stain for general histopathological architectures or Masson’s trichrome stain for collagen fibers. The expression profile of psoriasis-associated specific genes was determined using Real-Time PCR analysis. As a result, topical application of IMQ resulted in a significant increase of mean total and epithelial (epidermis) thicknesses, the number of inflammatory cells infiltrated in the dermis, and the decrease of dermis collagen fiber occupied regions in the ear tissues of IMQ and IMQ plus vaseline treated groups when compared to the intact control group. A significant increase of epithelial thickness and number of inflammatory cells infiltrated in the dermis of dorsal skin tissues were also noticed in IMQ and IMQ plus vaseline treated groups as compared to the intact control group, suggesting classic IMQ-induced hypersensitive psoriasis. IMQ-induced hypersensitive psoriasis related histopathological changes to the ear and dorsal skin tissues were significantly inhibited by the treatment of a standard drug clobetasol and SEL001. Further, mRNA expression analysis indicated a significant increase in gene expression levels of pro-inflammatory cytokines, including IL-19, IL-17A, and IL-23 in IMQ and IMQ plus vaseline treated groups than that of the control. Clobetasol and SEL001 treated groups resulted in a lower gene expression level of IL-19, IL-17A, and IL-23 as compared to IMQ and IMQ plus vaseline treated groups. These results enforce that SEL001 could be a novel treatment for psoriasis and an alternative to other drugs that pose a number of side effects on the skin.

## Introduction

The skin is one of the vital organs of the body that serves as a barrier from the outside environment. Psoriasis is a chronic skin disorder with unknown trigger, which is characterized by inflammation, thickening, and abnormal epidermal proliferation with up to 4% of prevalence in the general population (Griffiths and Barker, 2007). The pathogenesis of psoriasis is multifactorial and, as a perpetual incendiary skin issue, irregularity amongst pro- and anti-inflammatory mediators may play a vital part in the development and progression of this skin disorder (Li et al., 2016). Due to its incomplete etiology, there is still no permanent cure; however, genetic predisposition and environments stimuli might have a disease causing role. While the pathogenesis of psoriasis is not fully elucidated, it is extensively putative that pro-inflammatory cytokines have a key role in both development and maintenance of psoriatic lesions. A number of studies have reported the presence of immune-derived cytokines in psoriatic skin lesions and serum, suggesting their role in the pathogenesis of psoriasis (Kagami et al., 2010; Res et al., 2010; Zhang et al., 2010; Mudigonda et al., 2012; Tokura, 2012; Yoo et al., 2012; El Malki et al., 2013; Wang et al., 2013). Specifically, tissue alterations seen in psoriasis are driven by the exaggerated production of pro-inflammatory cytokines and the lesion development is critically dependent on IL-23 and IL-17 (Lee et al., 2004; Piskin et al., 2006; van der Fits et al., 2009). One of the pro-inflammatory cytokines, IL-19 has a key role in psoriatic pathogenesis and upregulates in psoriatic lesions during disease progression (Li et al., 2005), thus, controlling the level of these pro-inflammatory cytokines is necessary to treat this chronic disease.

The effect of the concurrent presence of IL-17 and IL-19 in psoriasis lesions might have impact on various levels of the pathogenic progression. The two cytokines upregulate the expression of β-defensins and S100A proteins thereby increases the antibacterial competence of keratinocytes. Moreover, their role in impeding infections may uphold the process to infiltrate immune cells to skin and increase the inflammation (Wolf et al., 2008). The process could be further regulated by tight coordination of IL-19 and IL-17 which in turn could attract particular T-helper type 1-cells, dendritic cells and neutrophilic granulocytes thereby directly increasing the secretion of chemokines. In addition, IL-19 and IL-17 promote the maintenance and effector function of T helper type 17-cells synergistically through the production of several mediators. Of note, T helper type 17-cells are critical for pathogenicity, IL-23 drives their proliferation, survival, and cytokine production (Ouyang et al., 2008). In addition, IL-19 and IL-17 are known to induce other interleukins which contributes to the psoriasis typical epidermal alterations. Topical application of IMQ, a TLR7/8 ligand and potent immune activator, can induce and exacerbate psoriasis, a chronic inflammatory skin disorder (van der Fits et al., 2009), and has been used as activator for valuable psoriasis animal model (Qin et al., 2014; Bai et al., 2016). Additionally, psoriasis may be linked with genuine comorbidities, for example, metabolic disorder and cardiovascular sickness, likely mirroring a systemic provocative part of the infection (Perera et al., 2012). At present, there are a number of temporary treatments for psoriasis ranging from tropical medicine, heliotherapy, and systematic to biological treatments (Gustafson et al., 2013). Nevertheless, the outcome of these treatments may result in severe side effects, thus, demands a much safer treatment to render this disease.

Probiotics are known as beneficial microorganisms that, when administered in adequate amounts, confer health benefits to the host (Joint FAO/WHO, 2001). The effect of probiotics on the skin could be mediated by the modulation of both the innate and the adaptive immune responses in the host. Modulation of an immune system is one of the beneficial effects of probiotics in human health. Recently, we reported that oral administration of heat-killed *Lactobacillus sakei* proBio65 inhibited immunoglobulin E-mediated histamine and β-hexosaminidase in NC/Nga mice, suggesting that *L. sakei* has an inhibitory effect on atopic dermatitis-like skin lesion (Park et al., 2008; Kim et al., 2013). Oral administration of *L. casei* has been seen to reduce antigen-specific skin inflammation by controlling the size of the CD8+ effector pool (Chapat et al., 2004). In addition, *L. casei* DN-114001 alleviates T-cell mediated skin inflammation (Hacini-Rachinel et al., 2009). Recently, *L. pentosus* GML-77 has been seen to inhibit skin lesions in IMQ-induced psoriasis in mice (Wu et al., 2017).

Use of probiotics and their cellular components in preventive medicine to maintain a healthy function is well documented (Matsumoto et al., 2005; Tojo et al., 2014). Probiotics have been proposed as therapeutic agents in various pathological conditions, including intestinal chronic inflammation, atopic dermatitis and gut homeostasis (Park et al., 2014; Tojo et al., 2014; Plaza-Díaz et al., 2017). Previously, we confirmed the therapeutic potential of SEL001 like products of plant and microbial origin *in vitro* and *in vivo* using a diabetic animal model (Shukla et al., 2011; Bajpai et al., 2016). Various studies have also shown that probiotics, such as *L. sakei* probio65 exhibit a wide range of pharmacological effects such as anti-atopic dermatitis in animals and humans (Park et al., 2008, 2014; Kim and Pyo, 2012; Kim et al., 2013, 2015c).

Although a number of studies have emphasized the therapeutic role of probiotics in various inflammatory conditions, there is no report available on the anti-inflammatory effects of SEL001 of probiotic origin in a psoriasis model of inflammation. Since the IMQ-induced psoriasis model highly resembles human psoriasis lesions (van der Fits et al., 2009), in this study, we investigated the effect of topical application of SEL001, a probiotic product isolated from *L. sakei* proBio65 in an IMQ-induced psoriasis mouse model.

## Materials and Methods

### Preparation of SEL001

To prepare the ethanolic extract of *Lactobacillus sakei* Probio65 hereby called SEL001, 48 h grown culture of strain *L. sakei* probio65 was mixed with ethanol (1:2 ratio) and the flask containing mixture of bacterial culture and ethanol was incubated at 150 rpm for 6 h at room temperature. Following the incubation, the mixture was centrifuged (8,000 *g* for 10 min) and the cell pellet was discarded. Resulting supernatant was filter sterilized and subjected to freeze drying until get a dry-powdered material, which was served as SEL001.

### Chemical Composition Analysis of SEL001

Gas chromatography–mass spectrometry (GC–MS) was explored to identify the chemical composition of SEL001. Briefly, dried SEL001 derived from the probiotic strain *L. sakei* probio65 was dissolved in 95% v/v methanol and two microliter of this solution was injected for GC-MS analysis. The protocol was followed as reported by Ravi et al. (2013).

### Animals

Thirty ICR mice of 6 weeks age were occupied from Samtaco Bio Co. (Korea), and were kept in cages at constant levels of temperature and humidity on 12 h light/dark cycles. The animals were acclimatized for 1 week and their backs were shaved using pet electric shaver (GSAK CO., LTD., Korea). The animals had free access to water and feed throughout the trial. Animal management and institutional approval for research protocols were supported and approved by the Animal Ethical Committee of the Yeungnam University (YNU-ANETCOMM-2016-00123), Gyeongsan, Korea.

### IMQ-Induced Psoriasis Model and Treatment

The mice were divided into five groups; a control group, an IMQ group, an IMQ+vaseline group, an IMQ-clobetasol group, and an IMQ+SEL001. Each group contains 6 mice and were housed as 2 mice per cage.

In this study a total of four topical treatments were used such as, IMQ, vaseline, clobetasol, and SEL001. IMQ was used to induce psoriasis like skin inflammation in mouse mode. Further, the treatment groups received vaseline, clobetasol and SEL001 topically 1 h before topical application of IMQ. Clobetasol propionate, a corticosteroid drug is used to treat various skin diseases, including psoriasis, therefore, it was used a gold standard, and SEL001 was used as a test sample against IMQ-induced psoriasis. Except for the control group, all other groups received a daily dose of 62.5 mg of 5% IMQ cream/cm^2^ (Aldara; MEDA AS) applied their backs and 20 mg on the right ear once a day for six consecutive days as previously described (van der Fits et al., 2009; Nadeem et al., 2015; Rather et al., 2016). Further, IMQ+vaseline group received a daily dose of 62.5 mg of 5% IMQ cream plus vaseline cream (80 mg/cm^2^ on back and 20 mg on the right ear); IMQ plus clobetasol group received a daily dose of 62.5 mg of 5% IMQ cream plus clobetasol (80 mg/cm^2^ on back and 20 mg on the right ear), and IMQ plus SEL001 group received a daily dose of 62.5 mg of 5% IMQ cream plus SEL001 (50 mg/cm^2^ on back and 10 mg on the right ear).

### Scoring of Skin Inflammation Severity

On days 0, 2, 4, and 6, all animas were assessed using 2 elements of the Psoriasis Area Severity Index (PASI), to consign a score of 0–4 (0, none; 1, moderate; 3, severe; 4, very severe) for both erythema and scaling parameters. Further, back skin and ear thickness were measured by using electronic caliper (Shenzhen Liweihui Technology Co., Ltd., China). After day 6, animals were euthanized and the shaved skin area of the back and ear was excised. Two lesions of the skin of each mouse were taken for histological and mRNA expression analysis.

### Histological Process

Approximated regions of an individual ear and dorsal skin tissues were sampled and they were crossly trimmed. All trimmed ear and dorsal skin tissues were re-fixed in 10% neutral buffered formalin (NBF), again for 24 h. After paraffin embedding, 3–4 μm sections were prepared. Representative sections were stained with hematoxylin and eosin (HE) for general histological architectures or Masson’s trichrome (MT) for collagen fibers in the dermis of the ear and dorsal back skin tissues (Kim et al., 2015a; Bai et al., 2016). The histological profiles of individual cross trimmed ear and dorsal skin tissues were observed under a light microscope (Model Eclipse 80*i*, Nikon, Tokyo, Japan). Histological evaluation was performed on the two fields in each part of the ear and dorsal skin tissues; consequently, six histological fields of an ear and dorsal back skin tissues in each group were considered for further histomorphometric analysis. The histopathological analysis was done by the histopathologist who was unaware of group distribution. To observe more detailed changes, mean total and epithelial thicknesses of ear and dorsal skin tissues (μm), mean numbers of inflammatory cells infiltrated in dermis of ear and dorsal skin tissues (cells/mm^2^ of dermis) and mean collagen fiber occupied regions in dermis of ear and dorsal skin tissues (%/mm^2^ of dermis) were calculated using a computer-based automated image analyzer (*i*Solution FL ver 9.1, IMT *i*-solution Inc., Vancouver, QC, Canada) according to our previously established methods (Qin et al., 2014; Kim et al., 2015b; Bai et al., 2016). At least five repeated measurements were considered to calculate each mean histomorphometric value, whenever possible, in this histopathological evaluation.

### Statistical Analysis

The data obtained were analyzed by one-way ANOVA test followed by least significant differences (LSD) multi-comparison test to determine which pairs of group comparison were significantly different. Differences were considered significant at *p* < 0.05.

### Quantitative Real-Time (RT) Reverse Transcriptase Polymerase Chain Reaction (PCR)

The extraction of RNA was done using Trizol^TM^ reagent (Invitrogen, United States) following manufacturer’s instructions. The extracted RNA was diluted in RNase-free water and quantified using Nano Drop by measuring the absorbance at 260 and 280 nm. Further, 1 μg of RNA was converted to cDNA using Maxime RT premix (iNtRON Biotechnology). Transcription levels of genes were quantitatively determined using RT-PCR (Stratagene 246 mix 3000p QPCR System, Agilent Technologies, Santa Clara, CA, United States) by employing power SYBR green (Roche Diagnostics Gmbh, Mannheim, Germany). The PCR reactions for each sample were run in duplicate, and for every gene, the transcription levels were normalized with β-actin. The primers used in this study were purchased from Microgen (Korea).

## Results

### Chemical Composition of SEL001

The GC–MS analysis of ethanolic extract of a probiotic strain *L. sakei* probio65 (SEL001) resulted in the identification of various compounds which included organic acids, amino acids, phenols, imidazole derivatives, and sugar alcohols along with some other organic compounds. The major composition of the SEL001 contained D-lactic acid (33.86%), propanoic acid (16.65%), glycerol (16.6%), L-lactic acid (6.03%), *myo*-inositol (1.86%), 1,3-butandiol (1.35%), 4-(methylsulfanylphenyl) carbamic acid (1.32%), 3,6-dioxa-2,7-disilaoctane (1.31%), pyrrolo[1,2-1] pyrazine-1,4-dione (1.26%), and valine (1.12%).

### Erythema and Scaling

**FIGURE 1:**
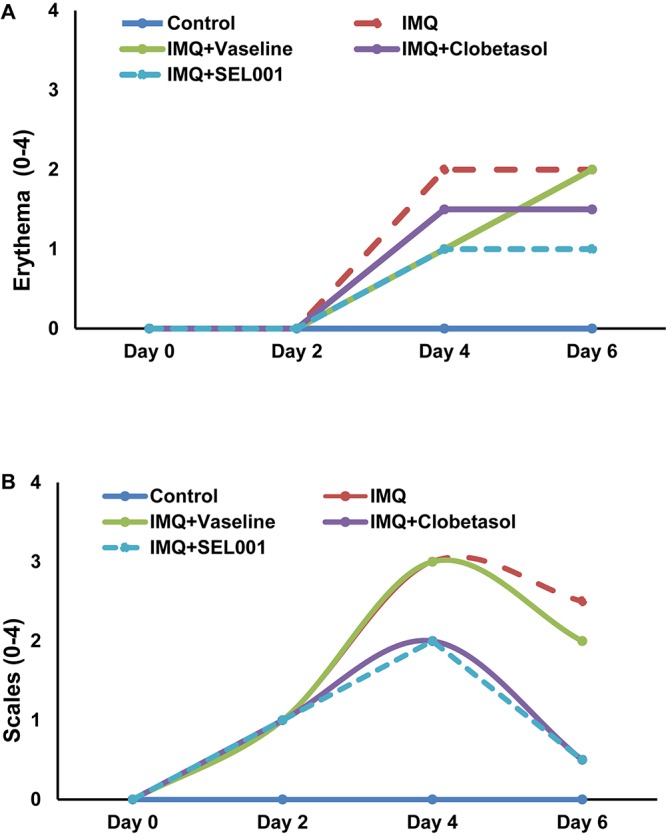
Erythema and scales score of the dorsal skin of the mice. The scoring was performed on 0 day, 2 day, 4 day, and 6 day using the Psoriasis Area Severity Index (PASI). **(A)** Erythema score and **(B)** scales score.

The severity of psoriasis during the 6-day trial was scored on 0, 2nd, 4th and 6th day following Psoriasis Area Severity Index (PASI). By applying IMQ on dorsal skin, psoriasis-like skin became apparent on second day onward. No significant changes in skin condition were seen on the second day in groups applied with vaseline, clobetasol or SEL001. However, from day three onward, erythema and scales were more visible. The erythema and scaling on mice back showed a dramatic increase in all groups as seen on day 4. However, a significant increase in erythema and scaling scores was seen in IMQ group when compared to control. In case of IMQ+vaseline group, a steady rate in erythema and slight decrease in scaling was observed from day 4 to day 6. In case of IMQ+clobetasol and IMQ+SEL001 the erythema scores were significantly less when compared to IMQ group (*p* < 0.01), and the scaling scores significantly decreased from day 4 to day 6+. Nevertheless, the erythema score in IMQ+clobetasol and IMQ+SEL001 was not completely normalized when compared to control group. The total mean scores for erythema and scales are shown in **Figures 1A,B**, from 0 day to day 6.

### Skin and Ear Thickness

**FIGURE 2:**
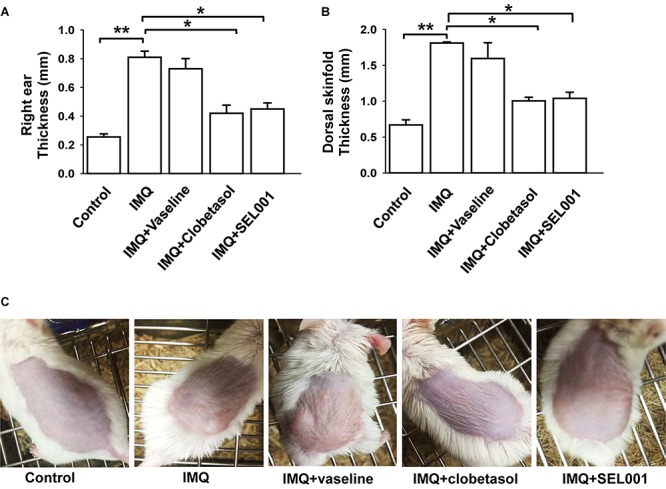
Caliper measurement and visual appearance of skin. **(A)** Skin fold thickness on the backs of the mice, **(B)** right ear thickness, and **(C)** visual appearance on day 6. Columns represent means ± standard deviation of skin/ear measurements day 6. ^∗∗^*p* < 0.001 or ^∗^*p* < 0.05 is significant, analyzed by one-way ANOVA test.

The ear thickness of right ear significantly increased in IMQ and IMQ+vaseline groups. In case of IMQ+clobetasol and IMQ+SEL001, the ear thickness was significantly reduced (**Figure 2A**). In addition, similar results were seen in dorsal skin thickness. The dorsal skinfold thickness of the mice in IMQ group and IMQ+vaseline showed a significant increase by topical application of IMQ treatment as compared with the intact control group (*p* < 0.01). The skinfold thickness in IMQ+clobetasol and IMQ+SEL001 group was significantly reduced compared to the IMQ group (*p* < 0.01) (**Figure 2B**). **Figure 2C** shows the visual appearance of IMQ-induced skin after 6 days of treatment. The skinfold thickness and ear thickening in IMQ+clobetasol and IMQ+SEL001 were not completely normalized when compared to control group. Nevertheless, the effect of clobetasol and SEL001 was promising.

### Histological Analysis

**FIGURE 3:**
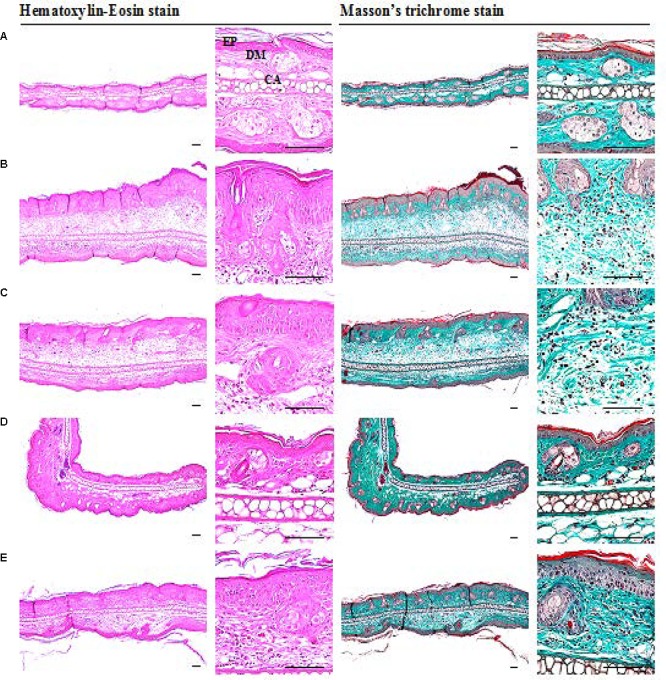
Histopathological images of ear tissues of IMQ-induced psoriasis mice. **(A)** Control, **(B)** IMQ, **(C)** IMQ+vaseline, **(D)** IMQ+clobetasol, and **(E)** IMQ+SEL001. A noticeable increase of total and epithelial (epidermis) thicknesses, inflammatory cell infiltrations in the dermis, and decreases of dermis collagen fibers were observed in ear tissues of IMQ and IMQ+vaseline as compared with intact control, respectively. However, these IMQ-induced hypersensitive psoriasis related histopathological changes on the ear tissues were significantly inhibited by treatment of test material SEL001 and reference drug clobetasol in the current histopathological inspection. No meaningful histopathological changes in the ear tissues were demonstrated in IMQ+vaseline as compared with those of IMQ control [IMQ = Imiquimod; EP = Epithelium/Epidermis; DM = Dermis; CA = Ear cartilage].

**FIGURE 4:**
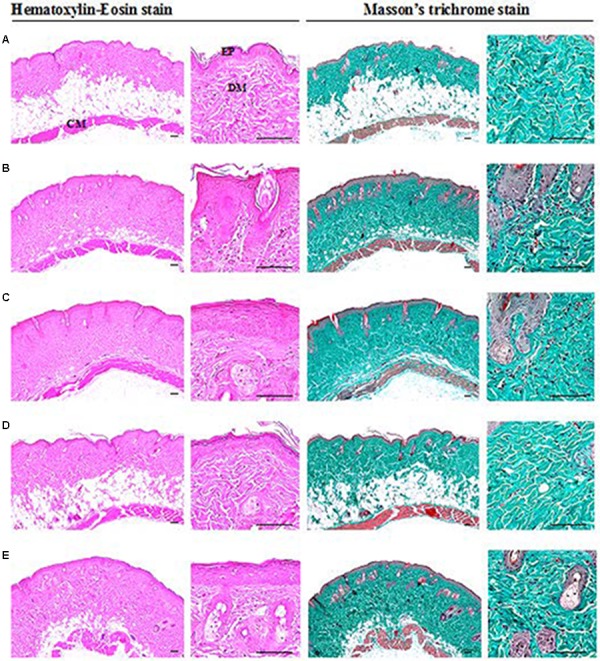
Histopathological images of dorsal skin tissues of IMQ-induced psoriasis mice. **(A)** Control, **(B)** IMQ, **(C)** IMQ+vaseline, **(D)** IMQ+clobetasol, and **(E)** IMQ+SEL001. Increase of dorsal skin epithelial thickness and inflammatory cell infiltrations in dermis of dorsal skin were noticed in IMQ control and IMQ+vaseline as compared with intact control, respectively. But no significant changes on the dorsal back skin total thicknesses and collagen fiber occupied regions in the dermis were demonstrated in IMQ control and IMQ+vaseline as compared with intact control and no significant histopathological changes in the dorsal skin tissues were demonstrated in IMQ+vaseline as compared with those of IMQ control. These IMQ-induced hypersensitive psoriasis related histopathological changes on the dorsal skin tissues were significantly inhibited by treatment of SEL001 and clobetasol.

A significant increase in epithelial thickness and number of inflammatory cells infiltrated in the dermis of ear tissues were observed in IMQ and IMQ+vaseline group compared to control group (*p* < 0.01), as shown in **Figure 3**. In addition, a significant increase in dorsal skin epithelial thickness and the number of inflammatory cells infiltrated in dermis of dorsal skin were also noticed in IMQ group and IMQ+vaseline group as compared with control group (*p* < 0.01), as shown in **Figure 4**. Moreover, no significant changes in total dorsal skin thickness and collagen fiber occupied regions in the dermis were observed in the IMQ group and IMQ+vaseline group as compared with the control group, and no significant histopathological changes in the ear and dorsal skin tissues were observed in the IMQ+vaseline group as compared with those of IMQ group. These IMQ-induced hypertensive psoriasis related histopathological changes in both ear and dorsal skin tissues were significantly inhibited in IMQ+clobetasol and IMQ+SEL001 groups (*p* < 0.01) as shown in **Tables 1**, **2**.

**Table 1:** Histomorphometrical analysis of ear tissues from control or IMQ-induced psoriasis mice.

Groups	Total thickness (μm)	Epithelial thickness (μm)	Inflammatory cell numbers (cells/mm^2^ of dermis)	Collagen occupied regions (%/mm^2^ of dermis)
Control	228.70 ± 23.62	20.14 ± 2.67	21.83 ± 3.54	66.92 ± 5.43
IMQ	651.42 ± 133.75^a^	84.97 ± 13.00^a^	566.50 ± 125.43^a^	42.56 ± 4.54^a^
IMQ+vaseline	653.42 ± 104.38^a^	83.30 ± 13.78^a^	550.17 ± 135.19^a^	41.65 ± 6.66^a^
IMQ+clobetasol	343.09 ± 86.69^bcd^	34.43 ± 10.71^bcd^	156.83 ± 38.59^bcd^	60.88 ± 1.07^cd^
IMQ+SEL001	426.78 ± 90.28^acd^	46.16 ± 13.00^acd^	286.00 ± 71.55^acd^	53.56 ± 6.61^acd^

*Values are expressed as mean ± SD of six ear histological fields. IMQ = Imiquimod, SEL001 = Test material; LSD = Least-significant differences multi-comparison. ^a^p < 0.01 and ^b^p < 0.05 as compared with control by LSD test; ^c^p < 0.01 as compared with IMQ by LSD test; ^d^p < 0.01 as compared with IMQ+vaseline by LSD test.*

**Table 2:** Histomorphometrical analysis of dorsal skin tissues taken from control or IMQ-induced psoriasis mice.

Groups	Total thickness (μm)	Epithelial thickness (μm)	Inflammatory cell numbers (cells/mm^2^ of dermis)	Collagen occupied regions (%/mm^2^ of dermis)
Control	807.56 ± 104.99	24.30 ± 6.19	43.33 ± 10.44	68.91 ± 10.00
IMQ	797.82 ± 105.24	65.11 ± 5.47^a^	319.83 ± 139.20^d^	67.82 ± 11.00
IMQ+vaseline	814.14 ± 76.68	66.41 ± 10.91^a^	320.17 ± 116.10^d^	67.51 ± 11.54
IMQ+clobetasol	792.12 ± 91.95	34.44 ± 10.47^bc^	37.00 ± 8.44^ef^	69.07 ± 10.28
IMQ+SEL001	803.39 ± 78.65	44.54 ± 11.40^abc^	79.67 ± 17.01^def^	70.84 ± 10.42

*Values are expressed as mean ± SD of six ear histological fields. IMQ = Imiquimod, SEL001 = Test material; LSD = Least-significant differences multi-comparison. ^a^p < 0.01 as compared with control by LSD test; ^b^p < 0.01 as compared with IMQ by LSD test; ^c^p < 0.01 as compared with IMQ+vaseline by LSD test; ^d^p < 0.01 as compared with control by MW test; ^e^p < 0.01 as compared with IMQ by MW test; ^f^p < 0.01 as compared with IMQ+vaseline by MW test.*

### Gene Expression Analysis

**FIGURE 5:**
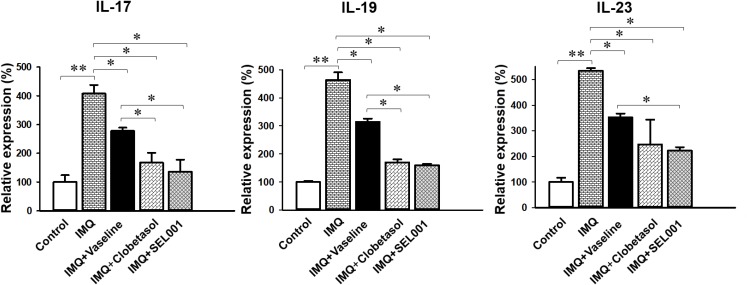
mRNA expression profile. The data is expressed as mean ± standard deviation, ^∗∗^*p* < 0.001 or ^∗^*p* < 0.05 is significant, analyzed by one-way ANOVA test.

Topical treatment of IMQ on the dorsal skin of mice showed an intense change in the gene expression level compared to the intact control, and/or mice treated with IMQ+clobetasol and IMQ+SEL001. The RNA expression analysis indicated an increase in gene expression of IL-19, IL-17A, and IL-23 in the IMQ group when compared with control group a pronounced drop of IL-19 was induced by clobetasol and SEL001 as seen in IMQ+clobetasol and IMQ+SEL001 groups. Similarly, a downregulation of gene expression level of IL-19, IL-17A, and IL-23 was seen in IMQ+clobetasol and IMQ+SEL001 groups compared to IMQ group and IMQ+vaseline group (**Figure 5**).

## Discussion

We have previously shown that *L. sakei* pro65 has a potent effect against atopic dermatitis both in the mouse model as well as in human clinical trial (Park et al., 2008; Woo et al., 2010). *Lactobacillus sakei* pro65 significantly reduced the IgE expression level in DNCB induced AD animal model (Kim et al., 2013). Furthermore, in our previous study, we showed *L. sakei* pro65 extract has antioxidant, anti-diabetic and tyrosinase inhibitory effects (Bajpai et al., 2016). Therefore, this study was designed to evaluate the effect of SEL001 on IMQ-induced psoriasis-like skin inflammation in a mouse model. Interestingly, chemical composition analysis of SEL001 confirmed the presence of various bioactive substances in SEL001, including organic acids, amino acids, phenols, imidazole derivatives, and sugar alcohols. As reported previously, these compounds or SEL001 like products of different origins containing phenolics and organic acids have been found to exhibit significant anti-inflammatory effects in various *in vitro* and *in vivo* models (Lee et al., 2006; Beh et al., 2017; Hearps et al., 2017). However, no effective agent has been developed against chronic psoriasis using the probiotic approach, which are known to be Generally Recognized As Safe (GRAS) in nature with no or less adversary effect for human application. Moreover, the SEL001 was found to contain important sugar alcohols, including *myo*-inositol. It has been reported that *myo*-inositol has been implicated with curing inflammatory disorders in animal mouse model (Claxson et al., 1990), suggesting that anti-inflammatory effect observed in this study might be mediated through *myo*-inositol. However, synergistic effects of other active components present in the SEL001 cannot be ruled out. As expected, the application of IMQ resulted in an increase in skin thickness and PASI score for both erythema and scaling. IMQ induced psoriasis model is considered as similar to human psoriasis (Leslie van der et al., 2009; Rather et al., 2016). Nevertheless, it was shown that IMQ induced skin lesions in humans differ to some extent from native psoriasis plaques (Vinter et al., 2015).

In the current histopathological inspection, skin protective effects of SEL001 were observed on the IMQ-induced hypersensitive psoriasis in a mouse model through well-documented histopathology-histomorphometric methods (Qin et al., 2014; Kim et al., 2015a,b; Bai et al., 2016). The results of representative histological profiles and histomorphometric analysis of the ear and dorsal back skin tissues are shown in **Figures 3** and **4**, respectively.

As reported previously, histopathologically, IMQ-induced hypersensitive psoriasis-like inflammation increased the epidermis hyperplasia and hypertrophy, and dermis inflammatory cell infiltrations in the ear and dorsal skin (van der Fits et al., 2009; Kim et al., 2015a; Bai et al., 2016), and similar results were observed in IMQ and IMQ+vaseline groups in this study. Significant increase of mean total and epidermal thicknesses, numbers of inflammatory cells infiltrated in the dermis, and decrease of dermis collagen fiber occupied regions were observed in the ear tissues of IMQ and IMQ+vaseline groups when compared with the control group. In addition, a significant increase of dorsal skin epithelial thicknesses and number of inflammatory cells infiltrated in the dermis of dorsal back skin were also noticed in IMQ and IMQ+vaseline groups as compared with control group, suggesting classic IMQ-induced hypersensitive psoriasis.

Moreover, no significant changes in the dorsal skin total thicknesses and collagen fiber occupied regions in dermis were demonstrated in IMQ group and IMQ+vaseline group as compared with control group. Also, in the present histopathological measurement, no significant histopathological changes in the tissue of both ear and dorsal skin were observed in IMQ+vaseline group as compared with those of IMQ group, suggesting that vaseline treatment did not show any effect on IMQ-induced hypersensitive psoriasis related histopathology in the ear and dorsal skin tissues. On the other hand, these IMQ-induced hypersensitive psoriasis related histopathological changes in both ear and dorsal skin tissues were significantly inhibited by the treatment of clobetasol and SEL001. Based on histopathological findings, SEL001 has obvious protective effects on the IMQ-induced hypersensitive psoriasis related histopathology in the ear and dorsal skin tissues. The decrease of collagen fibers in the dermis has been indicated edematous changes related to inflammations, as also considered in previous reports (Kim et al., 2015a,b). Decrease in the gene expression of IL-19, IL-17A, and IL-23 levels further confirms the potent effect of SEL001 on IMQ-induced psoriasis.

## Conclusion

In summary, in this study, the SEL001 ameliorates the severity of IMQ-induced psoriasis like skin inflammation in mice. Topical application of SEL001 significantly reduced the skin thickening, and improved the erythema and scaling scores. These findings were further supported by improvements in histoclinical symptoms. SEL001 significantly inhibited IMQ-induced skin epithelial thicknesses and dermis inflammatory cell infiltrations. Furthermore, treatment with SEL001 decreased the expression level of psoriasis-associated pro-inflammatory cytokines, such as IL-17A, IL-19, and IL-23, proposing that altogether these changes might be mediators of the positive effects of SEL001 observed in the psoriasis-like skin inflammation model used in this study.

## Author Contributions

IR and VB performed the experiments and analyzed the data. EB and WP helped in the interpretation of the data. JL and Y-HP participated in the design of the study and the interpretation of the data. IR and VB wrote the manuscript. All authors read and approved the manuscript.

## Conflict of Interest Statement

The authors declare that the research was conducted in the absence of any commercial or financial relationships that could be construed as a potential conflict of interest.
